# Supplementary material for: Active site specificity profiling datasets of matrix metalloproteinases (MMPs) 1, 2, 3, 7, 8, 9, 12, 13 and 14
Source: Data Brief. 2016 Feb 22;7:299–310. doi: 10.1016/j.dib.2016.02.036 (PMC4777984; doi:10.1016/j.dib.2016.02.036)
Supplement: Supplementary file 10 — Supplementary material [file mmc10.zip › WebPICS_hMMP1_G_1%/P3.html]

 

PICS results


|  |  |
| --- | --- |
| **P3\_A**  24 in 126 sites   19.0 %    effects > 10 perc. pnts.  (vice-versa in brackets)  P1\_Y: 11.1 (38.1)   P2prime\_L: 12.3 (18.5) |  |
| **P3\_P**  25 in 126 sites   19.8 %    effects > 10 perc. pnts.  (vice-versa in brackets)  P1\_N: -11.0 (-11.5)   P1prime\_I: 12.2 (12.2)   P1prime\_L: -25.2 (-10.1)   P2prime\_V: 13.7 (26.4) |  |
| **P3\_T**  11 in 126 sites   8.7 %    effects > 10 perc. pnts.  (vice-versa in brackets)  P1\_R: 11.1 (13.5)   P1prime\_C: 21.0 (28.8)   P2prime\_T: 10.3 (11.3)   P2prime\_V: 17.0 (14.4) |  |
| **P3\_V**  16 in 126 sites   12.7 %    effects > 10 perc. pnts.  (vice-versa in brackets)  P1\_A: 10.1 (14.6)   P1\_H: 13.2 (30.2)   P2\_L: 12.3 (12.3)   P2prime\_K: 25.5 (17.7)   P3prime\_K: 35.9 (57.3) |  |
